# Transplantation of hESC-derived hepatocytes protects mice from liver injury

**DOI:** 10.1186/s13287-015-0227-6

**Published:** 2015-12-12

**Authors:** Laia Tolosa, Jérôme Caron, Zara Hannoun, Marc Antoni, Silvia López, Deborah Burks, Jose Vicente Castell, Anne Weber, Maria-Jose Gomez-Lechon, Anne Dubart-Kupperschmitt

**Affiliations:** INSERM, U 1193, Hôpital Paul Brousse, Villejuif, F-94807 France; Unidad de Hepatología Experimental, IIS LA Fe, Valencia, S-46026 Spain; Univ Paris-Sud, UMR-S 1193, Villejuif, F-94800 France; DHU Hepatinov, Villejuif, F-94800 France; CIBERDEM, Centro de Investigacion Prıncipe Felipe, Valencia, S-46012 Spain; CIBERehd, FIS, Barcelona, S-08036 Spain

**Keywords:** Human embryonic stem cells (hESCs), Directed differentiation, Hepatocytes, Transplantation, Liver injury, Preclinical animal model, Cell therapy

## Abstract

**Background:**

Hepatic cell therapy has become a viable alternative to liver transplantation for life-threatening liver diseases. However, the supply of human hepatocytes is limited due to the shortage of suitable donor organs required to isolate high-quality cells. Human pluripotent stem cells reflect a potential renewable source for generating functional hepatocytes. However, most differentiation protocols use undefined matrices or factors of animal origin; as such, the resulting hepatocytes are not Good Manufacturing Practice compliant. Moreover, the preclinical studies employed to assess safety and function of human embryonic stem cell (hESC)-derived hepatocytes are generally limited to immunodeficient mice. In the present study, we evaluate the generation of hepatocytes under defined conditions using a European hESC line (VAL9) which was derived under animal-free conditions. The function capacity of VAL9-derived hepatocytes was assessed by transplantation into mice with acetaminophen-induced acute liver failure, a clinically relevant model.

**Methods:**

We developed a protocol that successfully differentiates hESCs into bipotent hepatic progenitors under defined conditions, without the use of chromatin modifiers such as dimethyl sulphoxide. These progenitors can be cryopreserved and are able to generate both committed precursors of cholangiocytes and neonate-like hepatocytes.

**Results:**

Thirty days post-differentiation, hESCs expressed hepatocyte-specific markers such as asialoglycoprotein receptor and hepatic nuclear factors including HNF4α. The cells exhibited properties of mature hepatocytes such as urea secretion and UGT1A1 and cytochrome P450 activities. When transplanted into mice with acetaminophen-induced acute liver failure, a model of liver damage, the VAL9-derived hepatocytes efficiently engrafted and proliferated, repopulating up to 10 % of the liver. In these transplanted livers, we observed a significant decrease of liver transaminases and found no evidence of tumourigenicity. Thus, VAL9-derived hepatocytes were able to rescue hepatic function in acetaminophen-treated animals.

**Conclusions:**

Our study reveals an efficient protocol for differentiating VAL9 hESCs to neonatal hepatocytes which are then able to repopulate livers in vivo without tumour induction. The human hepatocytes are able to rescue liver function in mice with acetaminophen-induced acute toxicity. These results provide proof-of-concept that replacement therapies using hESC-derived hepatocytes are effective for treating liver diseases.

**Electronic supplementary material:**

The online version of this article (doi:10.1186/s13287-015-0227-6) contains supplementary material, which is available to authorized users.

## Background

Hepatocyte transplantation has been proposed as an alternative to orthotopic liver transplantation for treatment of patients with acute liver failure (ALF) and metabolic disorders. Various clinical trials using hepatocyte transplantation have demonstrated partial improvement of liver function. However, the transplanted hepatocytes are unable to rescue patients due to the inadequate levels of engraftment [1, 2]. Moreover, there is an increasing shortage of viable and functional sources of human hepatocytes and the number of patients who die (15 %) while on the liver transplant waiting list has increased over the last few years. Recent advances targeted towards the differentiation of human embryonic stem cells (hESCs) or reprogrammed human induced pluripotent stem cells (hiPSCs) to various cell lineages offer significant promise for in vitro studies and as a source of viable cells for use in therapy. In the case of liver injuries or metabolic diseases, only a single cell type, the hepatocyte, is required [3]. Thus, the generation of an unlimited supply of these cells from pluripotent stem cells should be an important factor when translating stem cell biology into the clinic. Lastly, clinical data from patients with macular degeneration treated with hESC-derived retinal cells have demonstrated that hESCs may provide a potentially safe renewable and reliable source of cells for the treatment of various disorders [4]. Studies on liver development in model organisms have identified genes and signalling pathways vital for the formation of the hepatic lineage [5, 6] and, in recent years, a number of laboratories have reported various protocols that can successfully differentiate both hESCs and hiPSCs into hepatocyte-like cells by recapitulating liver development. The differentiation process is based on the initial induction of definitive endoderm [7, 8], followed by hepatic specification then differentiation into foetal hepatocyte-like cells (HLCs) [9–15] and, finally, further maturation into albumin-producing HLCs lacking nevertheless important features of adult primary hepatocytes. However, these approaches are based on culture media that contain serum and or chromatin modifiers (such as dimethyl sulphoxide or sodium butyrate), complex matrices such as Matrigel and/or the use of mouse embryonic fibroblasts as feeder cells. All of above are a source of unknown factors that could obscure the molecular mechanisms controlling human liver development or render the resulting tissues incompatible with future clinical applications. Over the last decade, our team and others have developed approaches using fully defined culture conditions required to generate HLCs from hESCs and hiPSCs [16–19]. Pluripotent stem cells were differentiated into a homogenous population of endoderm cells, which were then induced to differentiate further into hepatic bipotent progenitors, hepatoblasts, and then into foetal hepatocytes. It should be noted that all the approaches that have been developed on hESC differentiation into HLCs, including ours, have been conducted almost exclusively on a few US hESC lines, H9 being the most popular for generating HLCs [14, 20].

In the liver, heterotypic cell interactions between parenchymal cells and their non-parenchymal neighbours result in the regulation of differentiation and tissue proliferation in a three-dimensional microenvironment [21, 22]. The in vitro differentiation protocol does not reproduce this complex, three-dimensional, multicellular environment of the native liver. In this context, the engraftment and survival of HLCs in a native liver parenchyma should promote further maturation and long-term repopulation of transplanted cells. To date, the engraftment of human stem cell-derived HLCs has been described in a few models of immunodeficient mice with transgene-induced [13, 16, 23] or chemically induced [24–26] liver toxicity with low efficiencies. These data suggest that the transplanted cells were not responsive to the regenerative stimuli of host mouse liver and, therefore, not functional in vivo. However, the relationship between the level of HLC differentiation from pluripotent cells and the engraftment efficiency is not known. We previously demonstrated that foetal hepatoblasts isolated from human livers at an early stage of development (11–13 weeks of gestation) were able to engraft and exhibit in vivo mature functions such as CYP3A4 and a-glutathione-S-transferase activity [27–29]. Taken together, these data suggest that the transplanted stem cell-derived hepatic cells lacked various functions involved in their engraftment within the host parenchyma compared to foetal cells [23].

Utilising a hESC line derived in Centro de Investigacion Principe Felipe (CIPF) [30], we report a strategy for the efficient generation of functional human hepatocytes from VAL9 hESCs in animal-free conditions. Using sequential modulation of the different signalling pathways involved in the various developmental stages, we were able to generate cells that mimic functions of neonate hepatocytes (taken as reference) that also demonstrate key features of hepatocytes including the expression and activation of crucial cytochrome P450 enzymes and UDP glucuronosyltransferase 1A1 (UGT1A1). Finally, we demonstrate, for the first time, that the VAL9 hepatocytes (VAL9-HEP) were able to engraft and repopulate up to 20 % of the liver and rescue mice with acetaminophen-induced acute liver injury post-transplantation. These findings emphasize the potential value of these cells for use in liver cell therapy.

## Methods

### Cell culture

VAL9 hESCs were obtained from the Spanish National Stem Cell Bank (http://www.isciii.es/ISCIII/es) after approval of the InnovaLiv project by the following ethics committees: Spanish “Comision Nacional Medicina Regenerativa” on 21 May 2012 and French Agency of Biomedicine on 25 June 2012. VAL9 hESCs were cultured in feeder-free conditions on culture dishes pre-coated with 0.05 mg/ml Geltrex (Life technologies) in Nutristem medium (Biological industries) supplemented with 8 ng/ml fibroblast growth factor (FGF)2 (CellGenix) at 37 °C/5 % CO_2_ in animal-free conditions [30].

The hiPSC line was already established in the laboratory from human foreskin fibroblasts. hiPSCs were maintained on MEF feeders in DMEM/F12 medium supplemented with 20 % knockout serum replacement, 1 mM L-glutamine, 1 % non-essential amino acids, 0.1 mM β-mercaptoethanol and 4 ng/ml FGF2 at 37 °C/5 % CO_2_. Prior to differentiation, hiPSCs were plated on culture dishes pre-coated with Geltrex and maintained in the same conditions as VAL9 hESCs for a few passages before starting differentiation.

### Hepatic differentiation

Hepatic differentiation of hESCs and hiPSCs was performed following a multistep protocol adapted from Hannan et al. [19] up to the hepatoblast stage. First, Val9 hESCs at 80–90 % confluence are subjected to a 1-day treatment with 100 ng/ml Activin A (CellGenix), 100 ng/ml basic (b)FGF (CellGenix), 10 ng/ml BMP4 (R&D Systems), 10 μM LY294002 (Cayman Chemical Company) and 3 μM CHIR99021 (Miltenyi Biotec). On day 2, cells are exposed to the same cytokines in the absence of CHIR, and finally, on day 3, cells are exposed to 100 ng/ml Activin A, 100 ng/ml bFGF to induce definitive endoderm (DE). The efficiency of induction of DE was then assessed by immunofluorescence for SOX17, FOXA2 and GATA binding protein 4 (GATA4) and flow cytometry for CXCR4. Only cell preparations with above 85 % of CXCR4-positive cells were used for further differentiation. Cells were then exposed to 50 ng/ml Activin A for 3 days and 4 additional days to 10 ng/ml BMP4 and 10 ng/ml FGF10 (Biovalley) to promote hepatic specification. The hepatoblast stage was then assessed by immunofluorescence of hepatocyte nuclear factor (HNF)4α, CK19 and alpha foetoprotein (AFP) and flow cytometry for epithelial cell adhesion molecule (EpCAM). Confluent hepatoblast cells were then passed 1 to 2 in the presence of hepatocyte growth factor (HGF) 50 ng/ml to collagen-coated dishes and cultured in hepatocyte culture medium (HCM; Lonza) in the presence of HGF (Peprotech) and oncostatin M (OSM; Peprotech) for 3 days. Finally, cells were maintained in HCM in the presence of 20 ng/ml HGF up to day 30. The percentage of asialoglycoprotein receptor (ASGR)-positive cells was used to validate the efficiency of differentiation. The differentiation was also determined by immunofluorescence of hepatocyte markers such as albumin (ALB) and HNF4.

### Immunofluorescence

Cells were fixed with 4 % paraformaldehyde for 15 minutes at room temperature and permeabilized with 0.5 % Triton X-100 for 15 minutes. They were then incubated in 3 % bovine serum albumin (BSA)-phosphate-buffered saline (PBS) for 30 minutes at room temperature. Primary antibodies were diluted in 1 % BSA-PBS, and incubated overnight at 4 °C. Secondary antibodies were diluted in 1 % BSA-PBS and incubated for 1 hour at room temperature. (See primary and secondary antibody dilutions and information in Additional file 1: Table S1). All the photographs were taken with a Leica HMR microscope (Leica Microsystems).

### Flow cytometry analysis

Cells were dissociated with cell dissociation buffer, and suspended in 3 % BSA-PBS. They were then incubated with SSEA4-PE, CXCR4-APC or EpCAM-FITC conjugated antibodies or in control isotypes for 30 minutes at 4 °C in the dark. Cells were then washed with PBS, centrifuged, and suspended in PBS-BSA 1 % for analysis. Cells were detected in FL2 and FL4 channels with an Accuri C6 flow cytometer (BD biosciences). Dead cells were eliminated with 7AAD staining (Beckman coulter A07704). For ASGR analysis, cells were incubated with anti-ASGR antibody for 30 minutes at 4 °C and then with the secondary antibody Alexafluor 488 in the dark for 30 minutes at room temperature. Cells were then washed twice and suspended in PBS-BSA 1 % for analysis in the Accuri C6 flow cytometer. Quantification of cell death after thawing was analyzed by 7AAD staining.

### RNA extraction and real-time quantitative PCR

Total RNA was extracted from hepatocytes using a commercial kit (Qiagen) following the manufacturer’s recommendations. The amount of isolated RNA was estimated by ribogreen fluorescence and its purity was assessed by the absorbance ratio 260/280 nm. Total RNA (1 μg) was reverse-transcribed and real-time quantified using SYBR Green I Master and the appropriate primers (Additional file 2: Table S2) in a LightCycler 480 instrument. In parallel, the mRNA concentration of human housekeeping β-actin was always analysed as an internal normalization control. The real-time monitoring of the polymerase chain reaction (PCR) and the precise quantification of the products in the exponential phase of the amplification were performed with the LightCycler Relative Quantification Analysis software (Roche Applied Sciences) in accordance with the manufacturer’s recommendations. Moreover, a positive sample with a stable ratio of target and reference cDNA (a calibrator) was included in each PCR run to normalize all the samples within one run and to provide a constant calibration point among several amplification runs.

### Lentivector production and transduction of VAL9-derived cells

The EF1α–green fluorescent protein (GFP) and CYP3A4-GFP lentivectors were constructed and produced by Vectalys. The apolipoprotein A-II (APOA-II)–GFP lentivector was constructed in the laboratory and produced by Vectalys.

On day 13 of hepatic differentiation, cells were washed once with PBS, and fresh HamF12/Williams (HPM) supplemented with HGF (20 ng/ml final concentration) was added. The lentivectors were used at a multiplicity of infection (MOI) of 10 and were incubated with cells overnight. The cells were then cultured following the normal protocol.

For transplantation experiments, the VAL9-HEP were transduced at day 28 of differentiation and subsequently injected 2 days later.

### Functional characterization of differentiated cells

Ureogenesis was assessed in the thawed cells by measuring the formation of urea from NH_4_^+^, according to [31]. The periodic acid-Schiff (PAS) staining system was purchased from Sigma-Aldrich. In order to assess the response to hormones, VAL9-HEP were incubated with insulin (10^−7^ M) and/or glucagon (10^−6^ M) for 24 hours prior to the assay. Culture dishes containing cells were fixed in 4 % paraformaldehyde. Further assay was under the manufacturer’s instruction.

The indocyanine green (ICG) uptake test was assayed by incubating differentiated cells in medium containing 1 mg/ml ICG for 60 minutes at 37 °C. Cells were then washed three times with media and fresh HCM is added. ICG release was evaluated 24 hours later.

Cytochrome P450 activities were assayed by differentiated cells with a cocktail mixture of substrates for five individual P450 enzymes: 10 μM phenacetin (CYP1A2), 10 μM diclofenac (CYP2C9), 10 μM bufuralol (CYP2D6), 50 μM chlorzoxazone (CYP2E1) and 5 μM midazolam (CYP3A4). After 24 hours of incubation at 37 °C, cell media were recovered and stored at −80 °C until analysis. Formation of the corresponding metabolites was quantified by high-performance liquid chromatography tandem mass spectrometry (HPLC/MS; Waters) as previously described [32].

UGT1A1 activity was assayed by incubating differentiated cells in medium containing 15 μM β-estradiol for 24 hours. Cell media were recovered and stored at −80 °C until analysis, The formation of the corresponding metabolite was measured by HPLC/MS, as previously described [33].

### Animals and induction of acute liver failure (ALF)

Animals were housed at the animal facilities of the Instituto de Investigación Sanitaria La Fe. All animals had free access to food and water in a temperature-controlled room with a 12-hour dark/light cycle. All the animals received human care and all the experimental protocols were approved by the Institutional Animal Ethics Committee (Comite Etico de Bienestar Animal) of the La Fe Hospital and performed in accordance with Spanish national and institutional regulations. Male NOD/SCID mice (4–6 weeks) were treated with 300 mg acetaminophen (APAP)/kg to induce ALF 3 hours prior to cell transplantation. ALF was evaluated by means of histological staining and determination of transaminases in the sera of treated animals.

### Transplantation of VAL9-HEP into mice with ALF

At day 30 of differentiation, VAL9-HEP were collected and injected into the spleen of NOD/SCID mice with ALF. Three hours after the injection of APAP, mice were anaesthetized with a sevoflurane/O_2_ mixture and the lower pole of the spleen was exposed. Animals received an intrasplenic injection of 1 × 10^6^ VAL9-HEP in 200 μl infusion medium within seconds. The control mice, which had also received APAP treatment, received an intrasplenic injection of only the infusion medium [20].

At different time points, mice were sacrificed under anaesthesia (sevoflurane/O_2_ mixture). Blood was collected and serum aliquots were protected from light and stored at −80 °C until analysis. Liver and spleen were collected and stored at −80 °C until the histological analyses. From each tissue specimen, serial sections (7 μm) were cut with a cryostat (Micron HM 505 N) for fluorescent microscopy.

### Evaluation of tumourigenicity of differentiated cells

In order to assess the tumourigenic potential of VAL9-HEP, different tissues were histologically analysed. For this purpose, hematoxylin-eosin staining was performed and samples were examined by a pathologist in search of any sign of tumourigenicity.

### Enzyme-linked immunosorbent assay (ELISA) analysis

The human ALB values secreted into the medium of VAL9 cells (prior to or after differentiation) and secreted into the sera of transplanted animals were determined by the Human Albumin ELISA Quantitation kit (Bethyl; http://www.bethyl.com) following manufacturer’s instructions.

### Evaluation of engraftment of VAL9-HEP

Several sections from different lobes were used for the evaluation of the engrafted cells. The number of GFP-hepatocytes around portal and centrolobular veins was counted and referred to the total number of hepatocytes.

### Cholangiocyte differentiation and biliary cyst formation

hESC were differentiated into cholangiocytes as described by Dianat et al. [34].

The biliary cysts were generated as follows: day 18 cells were detached with trypsin, centrifuged and resuspended in biliary differentiation media (BDM; William’s E/Ham F12 1:1, 10^−5^ M linoleic acid-albumin (Sigma L9530), 5 × 10^−8^ M 3,3′,5-triiodo-L-thyronine (Sigma T2752), 0.2 IU insulin, 6.10^−4^ M vitamin C (Boyer), 6 × 10^−4^ M human apo-transferrin (Sigma T5391), 1 mM sodium pyruvate (Gibco)). The cells were then added to a mixture of rat-tail type I collagen (BD Biosciences), Matrigel 40 % (BD Biosciences), HEPES (0.02 M) and NaHCO_3_ (2.35 mg/ml). A cell suspension containing 5000 cells was added to 24-well inserts (BD biosciences 353104) and incubated at 37 °C for 3 hours. BDM medium (1 ml) supplemented with 20 ng/ml HGF and 10 ng/ml epidermal growth factor (EGF) was added on top of the insert as well as in the well, and incubated at 37 °C/5 % CO_2_ up to 2 weeks. The cells were fed every other day and, after 2 weeks, the cells were fixed in 3 % paraformaldehyde and stained.

## Results

### The differentiation of VAL9 hESCs into hepatic progenitors

At day 0 of the differentiation protocol, the VAL9-hESC colonies were positive for the pluripotency markers octamer-binding transcription factor 4 (OCT4), homeobox transcription factor (NANOG), tumour resistance antigen 1–60 (TRA1-60), SSEA4 and SSEA3. The cells also exhibited a normal karyotype (Additional file 3: Figure S1A–D).

**Fig. 1:**
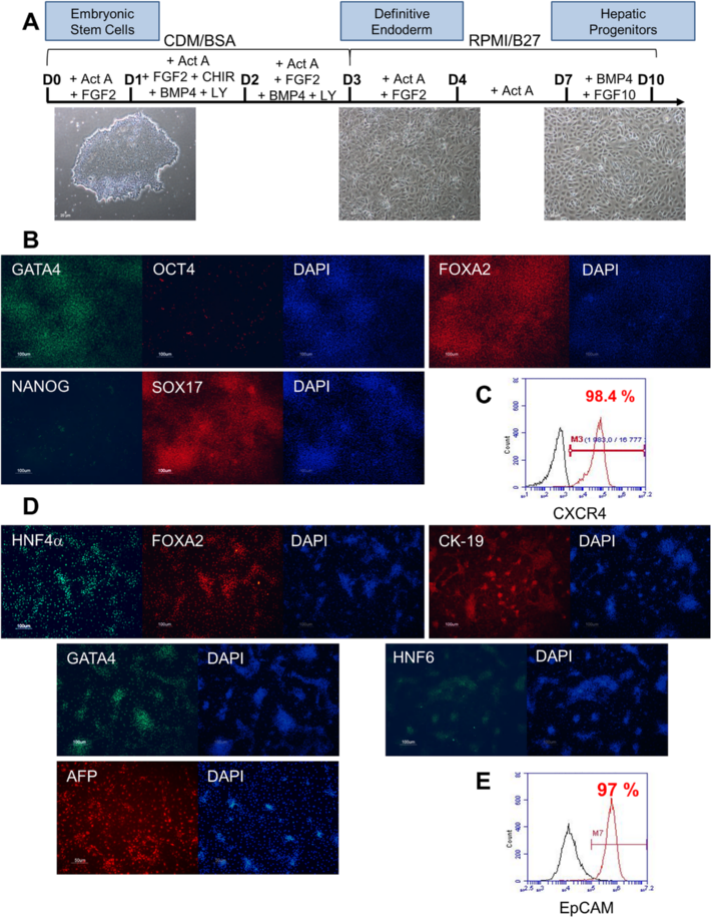
Differentiation of VAL9 hESCs into hepatic progenitors. **a** Stages of differentiation and phase contrast microscopy images showing corresponding cell morphology. **b** Representative images of immunofluorescence from definitive endoderm. Cells express GATA binding protein (GATA)4, SOX17, hepatic nuclear factor (HNF)3β and are negative for human octamer-binding transcription factor (OCT)4 and homeobox transcription factor (NANOG), two markers characteristic of undifferentiated cells. **c** Representative fluorescence-activated cell sorting (FACS) analysis of VAL9 at endoderm stage where 98.4 % cells were CXCR4-positive. **d** Representative immunofluorescence of hepatic progenitors. Cells express GATA4, HNF4α, HNF6, HNF3β, cytokeratin (CK)19 and AFP. **e** Representative FACS analysis of VAL9 at hepatic progenitor stage where 97 % cells were epithelial cell adhesion molecule (EpCAM)-positive.

We assessed the ability of a previously published protocol to generate hepatoblasts using the VAL9 cell line [19]. The VAL9 cells were subjected to a multistep differentiation protocol outlined in Fig. 1a. Clusters of hESC line were induced into definitive endoderm, followed by 6 days of hepatic specification, where the cells differentiated into hepatic bipotent progenitors. At day 5 more than 98 % of the cells expressed CXCR4 (Fig. 1c), a marker of DE. These cells were also found to be significantly positive for Sox17 and HNF3β, also markers of DE. Furthermore, less than 0.1 % of the cells expressed pluripotency markers (Fig. 1b). These data suggest that the VAL9 hESCs can be successfully differentiated into a homogenous population of endoderm cells. At day 11, the majority of the cells expressed CK19, GATA4, HNF3β, HNF6 and HNF4α, a master regulator of hepatic differentiation (Fig. 1d) and 97 % of the cells expressed EpCAM, a hepatoblast marker (Fig. 1e).

Transcriptional analysis of the in vitro differentiated progenitors demonstrated that these cells also expressed hepatic markers such as *HNF4α*, *AFP*, *FOXM1B*, and *LDLR* (Fig. 3f).

Cryopreservation and thawing procedures have been reported to have detrimental effects on the viability and function of primary human hepatocytes when compared to freshly isolated cells [35]. The successful cryopreservation of human hepatic progenitors that retain high viability, as well as the ability to be cultured and further differentiated, would allow for long-term banking of the cells required for subsequent research and clinical applications. We therefore assessed the ability of VAL9-derived progenitors to be thawed and cultured post-cryopreservation. As shown in Additional file 4 (Figure S2A–D) the hepatic progenitors maintained their cuboid morphology and were able to proliferate and express hepatic-specific markers such as AFP, HNF4α, FOXA2, CK19, EpCAM and AFP. Interestingly, the thawed cells also expressed claudin 1 (CLDN1), a co-receptor for hepatitis C virus (HCV) and a significant proportion of the cells also expressed CD81, another co-receptor for HCV. They also maintained a good viability (>80 %) along the post-thawing differentiation (Additional file 4: Figure S2D).

**Fig. 2:**
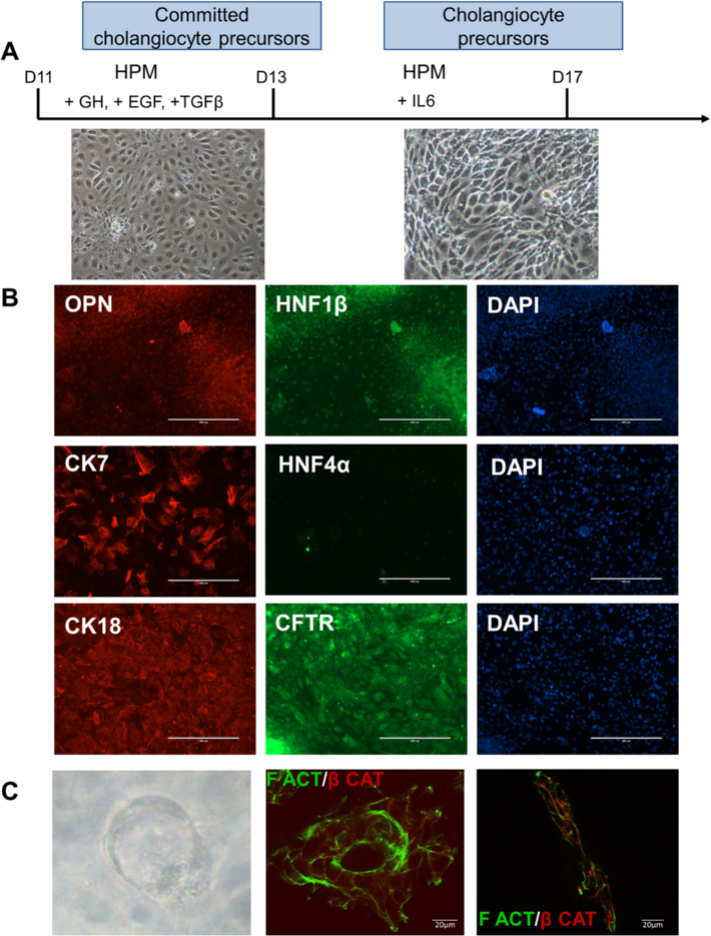
Differentiation of hepatoblasts into precursors of cholangiocytes. **a** Protocol and phase contrast images of cholangiocyte precursors. Hepatic progenitors were passaged at day 11 onto collagen 1-coated wells and grown for 2 days in HamF12/Williams (HPM), 20 ng/ml HGF, then for 2 days in HPM, 20 ng/ml HGF and 20 ng/ml epidermal growth factor (EGF). From day 16 to day 18 of differentiation cells were grown in a mixture of HPM/HCM. **b** Representative field of immunostaining of cholangiocyte precursor cells. Cells express osteopontin (OPN), hepatic nuclear factor (HNF1) 1β , cytokeratin (CK)7, CK18, cystic fibrosis transmembrane receptor (CFTR) and are negative for HNF4α, a hepatocyte transcription factor. **c** Representative phase contrast image (×40) of cysts obtained 1 week after seeding in matrigel at day 18 of differentiation (*left panel*). Immunostaining of cyst (*middle panel*) and tubule (*right panel*) showing the polarity of the cells in these structures. GH Growth hormone, IL Interleukin, TGF Transforming growth factor

As the hepatoblasts are bipotent progenitors, they are able to give rise not only to hepatocytes but also to cholangiocytes; we therefore investigated the capacity of VAL9-hepatoblasts to differentiate into committed cholangiocyte precursors. Treatment of hepatoblasts with GH/EGF, then interleukin-6 allowed the cells to reach confluence at around day 17 (Fig. 2a). This population of proliferating biliary-committed cells expressed osteopontin (OPN), a downstream target of NOTCH during normal liver development, HNF6 and HNF1β, whereas HNF4α expression was not detected, as shown by co-staining experiments with CK7/HNF4α (Fig. 2b). When grown in three dimensions these cells were able to generate ducts and tubules which showed polarity as demonstrated with F-actin and β-catenin staining (Fig. 2c).

### Differentiation of VAL9 hepatic progenitors into VAL9-HEP

**Fig. 3:**
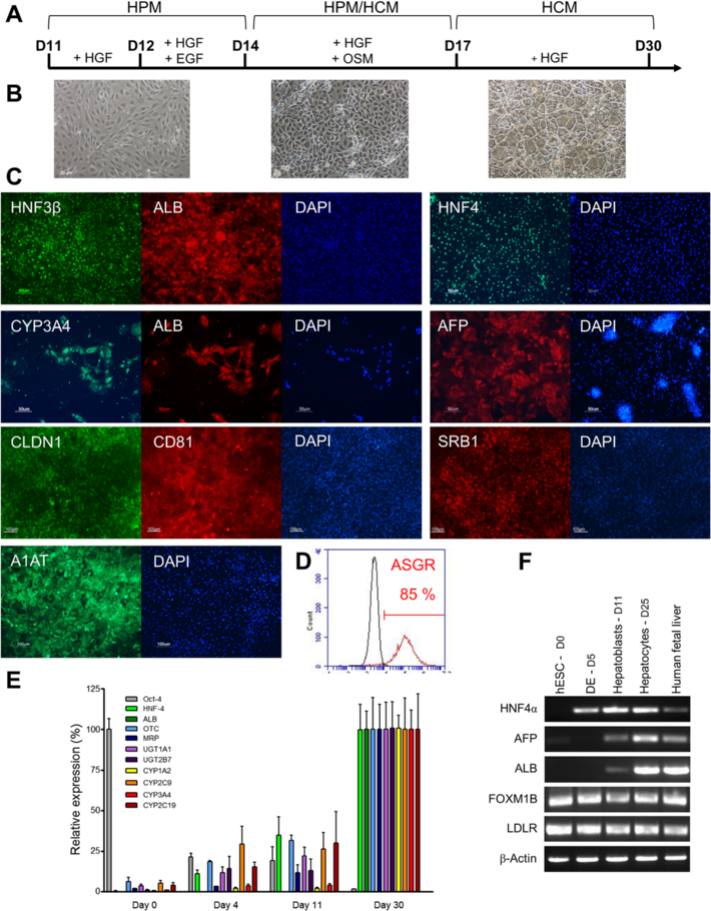
Differentiation of VAL9-hepatoblasts into hepatocytes. **a** Protocol and phase contrast images of hepatocyte differentiation. Hepatic progenitors were passaged at day 11 on collagen 1-coated wells and grown for 2 days in HamF12/Williams (HPM), 20 ng/ml hepatocyte growth factor (HGF), then for 2 days in HPM, 20 ng/ml HGF and 20 ng/ml epidermal growth factor (EGF). From day 16 to day 18 of differentiation cells were grown in a mixture of HPM/hepatocyte culture medium (HCM), HGF 10 ng/ml and oncostatin 10 ng/ml. Hepatocytes were generated after 10–12 additional days in HCM, 10 ng/ml HGF. **b** Hepatic morphology of VAL9-HEP during the differentiation protocol (11 to 30 days). **c** Representative field of immunostaining of VAL9-HEP. Cells express hepatocyte nuclear factor (HNF)Aα, alpha-1-antitrypsin (A1AT), albumin (ALB; *red*) and HNF3β (*green*), ALB (*red*) and cytochrome P450 3A4 (CYP3A4), alpha foetoprotein (AFP), claudin (CLDN1), scavenger receptor class B member 1 (SRB1) and cluster of differentiation (CD)81. **d** Representative fluorescence-activated cell sorting analysis of VAL9-HEP showing 85 % of asialoglycoprotein receptor (ASGR) a marker specific for differentiated hepatocytes. **e** Quantitative RT-PCR analysis at days 0, 4, 11 and 30 of differentiation. Data are represented as the percentage of expression in VAL9-HEP. **f** Representative transcript levels of hepatic cell markers on days 5, 11 and 25 of differentiation

Confluent hepatic progenitors were passaged at a ratio of 1:2 onto collagen 1-coated plates and allowed to further differentiate in the presence of HGF in medium supplemented with 10 ng/ml oncostatin for 3 days in addition to several other hepatic maturation factors (see Methods section) (Fig. 3a). At the end of this protocol, the differentiated cells exhibited characteristic hepatic morphology presenting a polygonal shape and round single or double nuclei (Fig. 3b). Immunostaining of the differentiated VAL9-HEP showed that the cells were positive for alpha-1-antitrypsin (A1AT) and ALB and the hepatic transcription factors HNF4α and HNF3β. Notably, ALB-expressing cells also expressed CYP3A4 (Fig. 3c). VAL9-HEP also expressed the entry cellular factors necessary for productive HCV infection, such as CD81 and CLDN1 (Fig. 3c). Fluorescence-activated cell sorting analysis revealed that 85 % of the cell population expressed ASGR, a cell surface receptor specifically expressed in the normal hepatocyte membrane (Fig. 3d).

The expression of liver-specific genes was assessed by quantitative reverse transcription PCR (Fig. 3e). The results showed the appearance of *HNF4α*, as expected, as early as day 11, and *ALB, MRP, UGT1A1, CYP1A2*, and *CYP2C9*, whereas expression of *OCT4* was abolished (Fig. 3e). The cells also expressed *ALB, LDLR* and the gene encoding the transcription factor FOXM1B as shown by RT-PCR (Fig. 3f).

### Characterization of VAL9-HEP functions in vitro

**Fig. 4:**
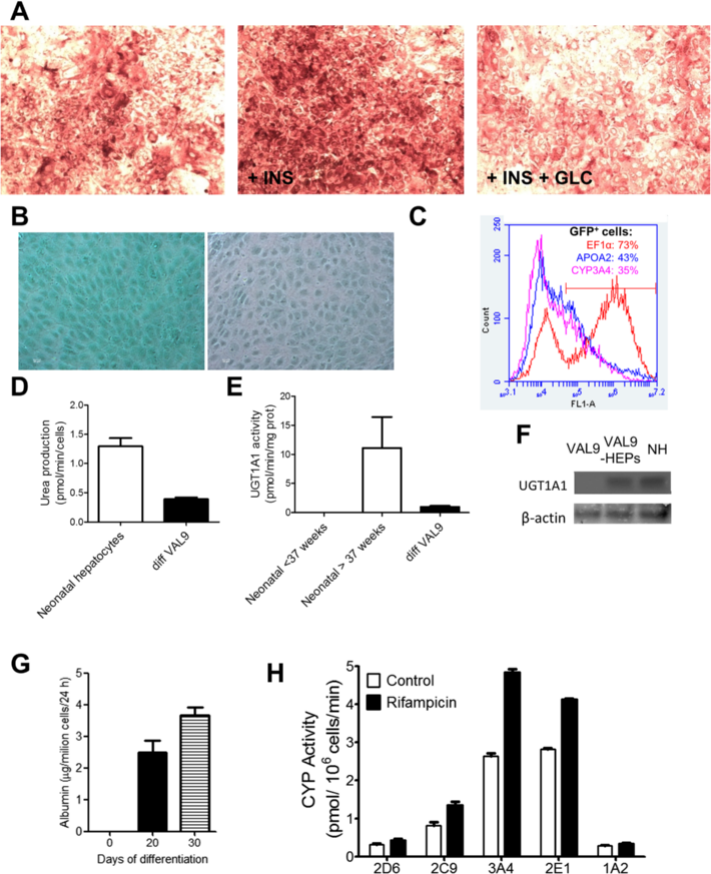
Functional characterization of VAL9-HEP in vitro at day 30 of differentiation. **a** Glycogen storage was assessed by PAS staining. Cells were incubated with insulin 10^−7^ M (INS) or INS 10^−7^ M + glucagon 10^−6^ M (GLC). **b** Cells were examined for uptake and excretion of ICG. **c** Differentiation of VAL9 hESCs was assessed by flow cytometry after transduction with lentivectors expressing green fluorescent protein (GFP) under the control of apolipoprotein A-II (*APOAII*) and cytochrome P450 3A4 (*CYP3A4*) promoters or *EF1α* promoter in control vectors. **d** Ureagenesis was assessed by measuring the formation of urea from NH^4+^ after incubation of the cells for 24 hours in the presence of NH4CL and by comparison to neonate hepatocytes. **e** UDP glucuronosyltransferase 1A1 (UGT1A1) activity was assessed by incubating VAL9-HEP with 15 μM β-estradiol for 24 hours and by comparison to neonate hepatocytes. **f** Western blot analysis showing expression of uridine 5’-diphospho-(UDP) glucuronosyltransferase 1 family, polypeptide A1 (UGT1A1) in VAL9-HEP and in neonatal hepatocytes (*NH*) using β-actin as loading control. **g** Human (h)ALB secreted to the media by VAL9-HEP after 20 and 30 days of differentiation. hALB was determined in the culture medium by ELISA. **h** CYP activity levels in VAL9-HEP were determined in cells exposed to 25 μM rifampicin and were compared to the control cells. Cells were incubated for 24 hours with a cocktail of specific substrates as previously described [55]. Activity values are expressed as pmol of the corresponding metabolite formed per minute and per million cells

VAL9-HEP demonstrated the ability to accumulate glycogen detected by PAS staining and these PAS-positive cells had the corresponding hepatocyte morphology. Moreover, the VAL9-HEP were responsive to hormones. Addition of insulin and glucose resulted in an increase in glycogen storage; by contrast, addition of glucagon to the cells resulted in a significant depletion of glycogen content (Fig. 4a). We also examined the cellular uptake and excretion of ICG, an organic dye that is taken up and subsequently eliminated specifically by hepatocytes. The cellular uptake was observed in VAL9-HEP in a very high percentage of cells and the majority of the ICG was excreted within a few hours and almost completely disappeared 24 hours later, indicating that a functional biotransforming system was generated in our VAL9-HEP (Fig. 4b).

An important function of hepatocytes is the ability to synthesize urea from ammonia and excrete it as urea. Therefore, we analysed the VAL9-HEP for urea excretion and compared the values to that of neonates [36]. We found that the VAL9-HEP values represented 28 % of mean neonatal values: 0.36 nmol/min/10^6^ cells and 1.30 ± 0.47 (0.76–2.19) nmol/min/10^6^ cells, respectively (Fig. 4d).

In contrast to foetal hepatocytes, the VAL9-HEP displayed UGT1A1 activity (1 pmol/mg/min) representing 10 % of that of newborn hepatocytes (Fig. 4e). In order to assess the specificity of the assay, the expression of UGT1A1 was also assessed by western blot. The VAL9-HEP expressed UGT1A1 when compared to undifferentiated VAL9, where the protein was not detected (Fig. 4f).

Another important function displayed by mature hepatocytes is serum protein production. ALB secretion was assessed using ELISA, and confirmed that post 20 and 30 days of differentiation ALB-secreting VAL9-HEP were generated, indicating their successful maturation (Fig. 4g).

The drug detoxification capability of VAL9-HEP was assessed by measuring the activities of the major cytochrome P450 enzymes responsible for the oxidative metabolism of drugs in the human liver (CYP1A2, CYP2C9, CYP2D6, CYP2E1 and CYP3A4). Differentiated cells displayed significant cytochrome P450 activity (Fig. 4f). An important property of hepatocyte function is the ability to respond to compounds able to induce the biosynthesis of an isozyme. The activity of CYP3A4 was significantly increased (100 %) when cells were treated with rifampicin when compared with untreated VAL9-HEP (Fig. 4h).

### Differentiation of hiPSCs

hiPSCs were differentiated using the same protocol as described above for VAL9 hESCs. As in VAL9 differentiation, hiPSC gave rise to a homogeneous population of endoderm cells expressing GATA4 and FOXA2 at day 5, then to bipotent progenitors expressing HNF4α and CK19 at day 11. They further differentiated into hiPSC-HEP expressing AFP and ALB (Additional file 5: Figure S3A–C). After 26 days of differentiation, hiPSC-HEP expressed hepatocytes markers, although the levels of gene expression were lower than in VAL9-HEP (Additional file 3: Figure S3E).

### VAL9-HEP rescue acetaminophen-induced ALF

A major challenge for stem cell-derived HLCs is their limited ability to mimic their in vivo counterparts. To assess the therapeutic potential of VAL9-HEP in vivo, we used the well-defined model of acetaminophen toxicity (APAP) in immunocompromised mice, which mimics ALF. This model was chosen because consistent hepatotoxicity has been shown in murine models and hepatocyte damage occurs at doses similar to those reported to provoke damage in human liver [37]. ALF is a multistep process that involves apoptosis followed by necrosis of hepatocytes in humans.

A dose of acetaminophen at 300 mg/kg body weight resulted in lethality in 50 % of the control animals 2 weeks after the administration of APAP.

**Fig. 5:**
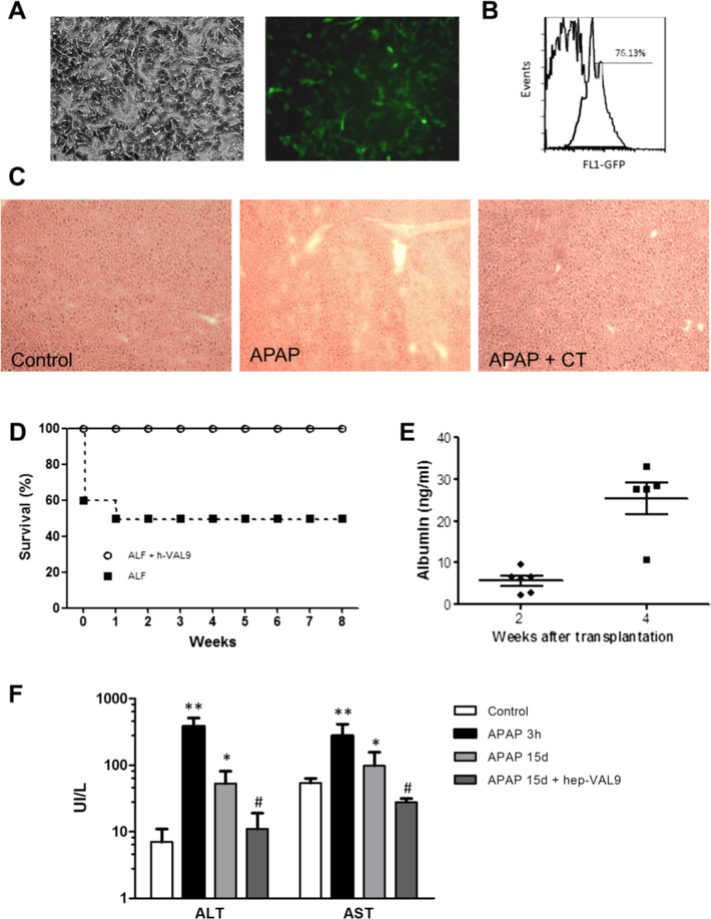
VAL9- HEP can rescue acetaminophen (*APAP*)-induced acute liver failure (*ALF*). **a** Representative images of transduced VAL9-HEP (phase-contrast and fluorescence) for green fluorescent protein (*GFP*). **b** Flow cytometry analysis of GFP expression from transduced VAL9-HEP 2 days after transduction with A1AT-GFP lentivector. The numbers indicate the percentages of GFP-positive cells in the analyzed gate. **c** Representative histopathology of livers of control, acetaminophen-treated mice and acetaminophen-treated mice after transplantation with VAL9-HEP. **d** Survival curves of immunodeficient mice (n = 10 in each group) that received intrasplenic cell transplantation with 1.0 × 10^6^ VAL9-derived hepatocytes. **e** Secreted ALB in the serum of transplanted animals at different time points. **f** Liver function test after infusion of acetaminophen then saline or VAL9-HEP at different time points. *p < 0.05, **p < 0.01 versus control; ^#^ *p* < 0.05 versus APAP-treated mice. *ALT* Alanine aminotransferase, *AST* Aspartate aminotransferase

Histological analysis indicated the presence of massive necrosis in the liver which became apparent as soon as 3 hours after APAP injection (Fig. 5b) and was concomitant with the release of alanine aminotransferase (ALT) and aspartate aminotransferase (AST) in the circulation (Fig. 5e).

To genetically trace the transplanted cells in vivo, VAL9-HEP were transduced in vitro with a GFP-expressing lentiviral vector under the control of the hepatic specific promoter of the human *A1AT* gene. Thus, GFP was expected to be expressed in the differentiated VAL9 cells only. As shown in Fig. 5a, 75 % of the transduced VAL9-HEP expressed GFP, suggesting that the majority of the transduced cells were differentiated hepatocytes.

VAL9-HEP transplantation resulted in a three- and fivefold reduction in AST and ALT values, respectively, when compared to control animals (Fig. 5f).

Fifty percent of the untreated control animals with ALF died within 2 weeks of transplantation, whereas all the animals which were transplanted with VAL9-HEP survived, indicating a survival advantage for those animals receiving cell therapy (Fig. 5c). Thus, the VAL9-HEP display sufficient detoxifying enzyme activity required to rescue the animals. Transplanted mice were sacrificed at three time points (2, 4 and 8 weeks) after transplantation. To verify that the transplanted hepatocytes homed towards the liver without migrating to other organs, we analysed the transplanted cell distribution in other organs such as the spleen and lungs 8 weeks after transplantation. No human cells were detected in any of the analysed organs as assessed by immunohistochemistry (IHC) against GFP (data not shown). IHC revealed that the liver displayed a normal histology with no sign of tumours (data not shown). The spleen, lung and kidneys were also normal. No signs of adenocarcinomas were visible in the peritoneum (data not shown).

### Engraftment of VAL9-HEP in APAP-treated mice

**Fig. 6:**
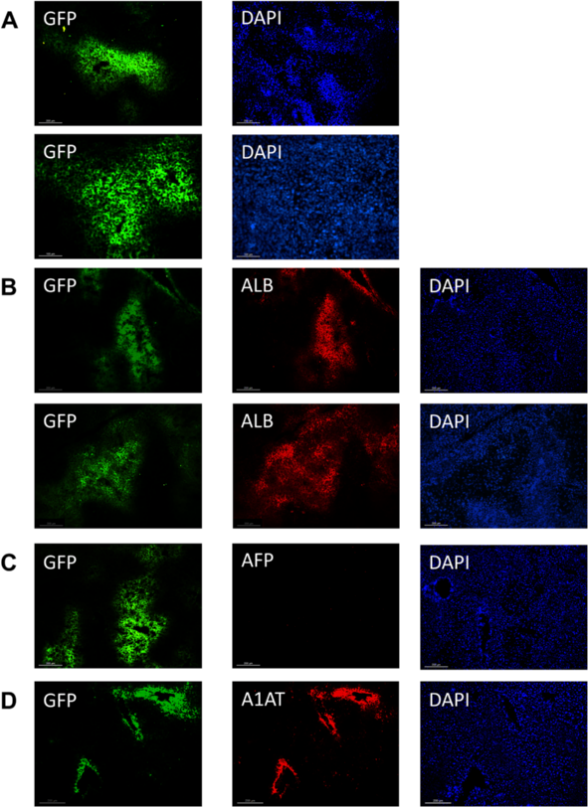
Engraftment of VAL9-HEP in the liver parenchyma of transplanted acetaminophen-treated mice. **a** Visualization of engrafted cells 30 days post-transplantation using immunohistochemistry (IHC) to detect green fluorescent protein (GFP). The clusters confirm proliferation of engrafted cells. **b** Representative IHC staining on serial sections showing co-expression of GFP and albumin (ALB). **c** Representative IHC staining on serial sections demonstrating downregulation of alpha foetoprotein (AFP). **d** Representative IHC staining on serial sections showing co-expression of GFP and alpha-1-anti-trypsin (A1AT)

In order to investigate whether the transplanted cells were engrafted within the livers of the recipient mice, we first used an antibody against GFP to detect the presence of human VAL9-HEP. Human cells were visible throughout the liver parenchyma in the form of clusters. This indicates that, in response to liver failure, the transplanted cells have not only engrafted but also proliferated (Fig. 6a). GFP-expressing VAL9-HEP were found in all the mice analysed (n = 8). By counting several sections from each mouse and different lobes we calculated that the percentage of liver repopulation ranged from 0.6 to 10.2 % of the liver parenchyma. Since 75 % of transplanted cells expressed GFP, the proportion of engrafted cells is underestimated.

Human ALB was then measured using ELISA in the sera of transplanted mice and control non-transplanted mice for 2 and 4 weeks post-engraftment (Fig. 5e). Human ALB was detected in every injected mouse, confirming the successful engraftment of VAL9-HEP.

In addition, the hepatic functions of the engrafted VAL9-HEP were identified using IHC; namely the detection of human ALB expression. A large number of positive cells co-expressing ALB and GFP were detected in the parenchyma at 4 weeks after transplantation confirming that VAL9-HEP were integrated and functional post-transplantation. We also performed co-immunostaining on sections of engrafted livers at day 30 and compared them to the VAL9-HEP used for the transplantation. Before transplantation a significant proportion of VAL9-HEP expressed AFP (Fig. 3). However, 4 weeks after transplantation all engrafted ALB-positive cells were negative for human AFP expression, demonstrating that it was downregulated as in endogenous hepatocytes (Fig. 6c). Non-transplanted control liver tissues were negative for human proteins (data not shown). Together, these results demonstrate that engrafted VAL9-HEP underwent maturation in situ.

## Discussion

We report the development of a new strategy to generate a homogenous population of hepatocytes. We used a new cohort of hESC, VAL9 cells, which were established in traceable conditions easily transposable to Good Manufacturing Practice (GMP) compatible conditions. We demonstrate that VAL9 hESCs can be efficiently differentiated, recapitulating the key stages of liver development, into viable hepatic cells with various hepatocyte-specific functions both in vitro and in vivo, where the hepatocytes were able to rescue mice with ALF. This was accomplished by the stepwise addition of defined factors, without the addition of mesenchymal or endothelial cells or any other liver cells that normally accompany hepatocyte development, nor serum or complex matrixes. However, although recombinant proteins such as fibronectin can be used (unpublished data), we utilized gelatin for cell differentiation up to hepatoblast stage and then collagen which is the matrix currently used for primary cell culture. Although in our experiments both of these matrix components were from animal origin, it is noteworthy that GMP-compatible, recombinant human collagen is now available as well as GMP-grade gelatin, suggesting that in the near future differentiation of pluripotent stem cells into hepatocytes will be possible under conditions appropriate for clinical applications. hiPSCs were also differentiated into hepatocyte-like cells following the same protocol used with VAL9-hESCs, highlighting the therapeutic potential of our approach.

At the progenitor stage, hepatoblasts could be cryopreserved and further differentiated into hepatocytes. They were also successfully induced to differentiate along the cholangiocyte lineage as previously reported [34], although conditions for further differentiation need to be improved.

At the hepatocyte stage, VAL9-HEP retain some characteristics of foetal hepatocytes, such as expression of AFP. However, the differentiated cells reproduce key features of mature hepatocytes, such as ICG metabolism. Due to the importance of maintaining blood glucose levels, the synthesis and degradation of hepatic glycogen, the storage form of glucose in the liver, are tightly regulated and the binding of hormones, such as glucagon, to cell receptors signals the need for glycogen to be degraded. Although the signalling pathways were not investigated, our data show that, upon the addition of glucagon, the amount of glycogen storage decreased in VAL9-HEP. It should be noted that fresh and thawed VAL9-HEP also exhibited the expression of receptors known to be involved in HCV infection.

Different strategies have been used to improve the differentiation of the hepatocyte-like cells (HLCs) in vitro*,* such as the use of special matrices, co-culture with stromal cells or purification of hepatoblasts [20, 38, 39]. However, to date, a protocol has yet to be developed resulting in the maturation of the HLCs at a comparable level to that of primary adult hepatocytes. This was reported by Baxter and colleagues after the extensive characterization of several hESC and iPSC lines including the evaluation of CYP activities [18]. To date, the expression of cytochrome P450 enzymes was mostly studied at the transcriptional level and CYP activities, when measured, were assessed by luminometry, which was misleading [18].

Drug-metabolizing enzymes are expressed at negligible or very low levels in the foetus. Recently, we have demonstrated the importance of both gestational and postnatal development for the maturation of CYPs in neonatal hepatocytes [36]. CYP2D6 and CYP1A2 activity was not detected in the younger neonatal hepatocytes [36]. Although CYP3A4 activity was low in VAL9-HEP, CYP2D6 and CYP1A2 activities were in the range of that detected in new-born hepatocytes, suggesting that the “adult” levels could be reached in transplanted cells after in vivo maturation.

Urea is formed within the urea cycle and represents the major end-product of ammonia detoxification in the liver. It is a good indicator of the degree of hepatocyte mitochondria preservation. VAL9-HEP were able to synthesize urea from ammonia at rates representing one-quarter to one-half of neonate values, indicating that this pathway is active in our cells [36]. The cells also displayed UGT1A1 activity, representing one-tenth of neonate values. In humans, UDP-glucuronosyltransferases (UGTs) are an important group of Phase II (conjugative) metabolizing enzymes that play a critical role in human health and disease. UGTs are involved in the metabolism and detoxification of numerous endogenous compounds and xenobiotic chemicals including therapeutic agents such as acetominophen [40]. The activity of UGT1A1, the major enzyme responsible for bilirubin glucuronidation, is not detected in the foetal liver [41]; it is induced after birth, which accounts for the onset of hyperbilirubinemia. Interestingly, type 1 Crigler-Najjar syndrome, a genetic deficiency in hepatic UGT1A1, is a metabolic disorder treated by hepatocyte transplantation.

Since transplantation of foetal stem or progenitor cells into livers of immunodeficient mice resulted in cell expansion and maturation [27, 28, 42, 43], we evaluated these properties after VAL9-HEP transplantation. It was reported that immature iPSC-derived HLCs (with a few cells weakly positive for CYP3A4) could engraft in Mu-uPA SCID transgenic mice expressing urokinase in the liver [44]. This model has been widely employed to produce an ideal chronic liver injury model used for transplanting primary hepatocytes and HLCs. Several studies suggest that uPA facilitates the engraftment and proliferation of transplanted hepatocytes. In liver regeneration, uPA activates plasminogen, which degrades the extracellular matrix to promote reorganization of the hepatic architecture [45]*.* In a model of CCL4-intoxicated animals, subpopulations of HLCs were transplanted after laser microdissection and pressure capturing, which selected for ICG high cells. This resulted in 10 % of ALB-expressing cells [26]. Although these models cannot be transposed to clinical situations, the data suggest that the maturity level of the pluripotent stem cell-derived hepatocyte-like cells plays a vital role in the efficiency of engraftment.

To assess the function of VAL9-HEP, we chose the mouse model of APAP-induced hepatotoxicity due to its clinical relevance. APAP overdose accounts for the majority of cases of drug liver injury resulting in fatal ALF [46]. It has become the most common cause of ALF in the United Kingdom and accounts for approximately half of ALF cases in the United States [47, 48]*.*

Our data show significant engraftment of VAL9-HEP in the liver parenchyma for at least 1 month. The efficiency of repopulation was approximately 15 %. Given that a mean of 75 % of hepatocytes expressed GFP prior to transplantation, efficiency of repopulation is underestimated and should more likely represent 18–20 %. Importantly, the engrafted cells also demonstrated in vivo maturation.

Moreover, it is imperative that therapeutically advantageous hepatocyte-like cells are safe (i.e. non tumourigenic), express hepatic-specific genes comparable to mature hepatocytes, and contribute to liver function in vivo as demonstrated by VAL9-HEP. The potential of hESCs and their differentiated progeny to generate spontaneous tumours is of particular concern with regards to their use in clinical applications. Several reports show tumour formation post-transplantation of hESC-derived cells despite pre-differentiation [10, 49–51] and demonstrate that the transplanted cells contain a number of undifferentiated hESCs [52]. On the other hand, additional studies have demonstrated that the transplantation of highly differentiated cells did not result in tumour formation [53, 54], thus suggesting that directing hESCs to an appropriate state is an important step for their safe and effective use in cell therapies. To this end, well-defined methods should be established to reduce the tumourigenicity of transplanted cells and a strict elimination of undifferentiated hESCs from transplanted cells [55, 56]. In our VAL9-HEP engrafted mice, we investigated the appearance of tumours at the time of sacrifice. No sign of tumour formation was evident in grafted livers or in other major organs. However, additional long-term studies are required to confirm the ultimate safety of VAL9-HEP.

We demonstrate in this study, for the first time, that engrafted human VAL9-HEP are able to rescue mice with ALF. This was evaluated by the significant decrease in AST and ALT and by the rescue of transplanted animals. The data suggest that the VAL9-HEP expressed sufficient levels of detoxification enzymes at the time of transplantation. Different models show a correlation between the number of infused cells and the percentage of repopulation; up to 2 to 7 M HLCs were infused per mouse [25, 26, 44]. We infused a significantly lower amount of cells, 1 million, which corresponds to 1–1.5 % of the mouse hepatocyte mass, which was enough to rescue half of the animals. Interestingly, hepatocytes derived from the HepaRG cell line were able to rescue CCL4-treated animals only when the cells were transduced with LXRα [57].

In humans, transplanting no more than 1–2 % of liver mass per cell infusion is recommended in order to avoid portal hypertension [58–60]. The scarce supply of donated cadaveric livers combined with the fact that mature hepatocytes display short-term survival, poor in vitro proliferation and tolerance to cryopreservation, results in limited transplantation options for patients, as well as use in other applications such as high-throughput drug screening. Clinical trials have been ongoing for years to assess the effects of foetal liver cells transplanted into patients with various liver diseases, in particular cirrhosis or inborn metabolic diseases [61]. Recently, a clinical trial was performed with freshly isolated biliary tree stem cells to treat patients with advanced cirrhosis. This procedure resulted in a 6- to 12-month improvement in both biochemical and clinical features [62].

Immunosuppression is an important issue in cell therapy strategies for liver diseases, but optimal regimens for inducing tolerance to transplanted liver cells are not well established. Even if hESC-hepatocytes could be antigen-matched to the recipient, immunosuppression would still be required, since hESCs express low levels of HLA class I antigens [63] and they are still subjected to immune system targeting. Therefore, this question will have to be addressed, but in a specific study using embryonic stem cells and an animal model from the same species.

The strategy that we describe here may address the problem of cell limitation, as it utilizes a renewable cell source. It offers the advantage of immediate availability and unlimited supply of functional donor hepatocytes for emergency treatments required by patients with ALF when an organ is not immediately available. In addition, unrestricted availability of donor hepatocytes could allow programmed and repeated treatment of patients with debilitating, liver-based metabolic disorders, which are not now considered candidates for organ transplantation, such as familial hypercholesterolemia and partial urea cycle disorders. The generation of hepatocytes also provides a potentially useful step toward the generation of hepatic organs. Tissue engineering of a hepatic organ will require the incorporation of hepatic niche cells, such as mesenchymal, stellate, endothelial cells and cholangiocytes, into cultures of stem cell-derived hepatocytes [64, 65].

## Conclusions

In summary, our strategy allows the stepwise differentiation in defined conditions of the new VAL9 hESC line into bipotent progenitors that are able to give rise to cholangiocyte precursors, then into neonate-like hepatocytes with detoxification activities. VAL9-HEP were able to successfully engraft and proliferate into mice livers suffering from acute failure in a clinically relevant model, resulting in a decrease of transaminases to control levels and the rescue of transplanted mice. Taken together, our data suggest that cell therapy using hESC-derived hepatocytes may be an effective treatment for liver diseases.

## Additional files

Additional file 1: Table S1. Antibodies used in this study. (PDF 48.5 KB) Additional file 2: Table S2. Primers used for RT-PCR of Human mRNAs. (PDF 79.6 KB) Additional file 3: Figure S1. Pluripotency of VAL9 hESCs. (**A**) Phase-contrast image of a representative VAL9 hESC colony. (**B**) Representative immunofluorescence staining for human OCT-4, NANOG and TRA1-60. (**C**) Fluorescence-activated cell sorting (FACS) analysis of the expression of stem cell specific surface markers: TRA1-81, SSEA-3, SSEA-4. (**D**) Karyotype analysis of VAL9 hESCs. (TIF 4911 kb) Additional file 4: Figure S2. Cryopreservation of VAL9-hepatoblasts. (**A**) Phase-contrast image of a representative VAL9-hepatoblasts at day 15 of differentiation before (fresh) and after cryopreservation (4 days after thawing). (**B**) Phase-contrast image of a representative VAL9-hepatoblasts at day 20 of differentiation (9 days after thawing). (**C**) Representative immunofluorescence staining for human cytokeratin (CK)19, hepatic nuclear factor (HNF)1α, alpha foetoprotein (AFP), FOXA2 ; and co-staining for human hepatic nuclear factor (HNF)4α/epithelial cell adhesion molecule (EpCAM), and for human claudin (CLDN)1/CD81. (**D**) Representative fluorescence-activated cell sorting (FACS) analysis for cell viability of thawed VAL9 cells at different time points after thawing as detected by 7AAD staining (TIF 5667 kb) Additional file 5: Figure S3. Differentiation of hiPSCs into hepatocytes. (**A**) Phase contrast microscopy images of cell morphology at different key steps of the differentiation protocol. (**B**) Representative immunofluorescence staining of definitive endoderm. Cells express GATA binding protein (GATA)4, and hepatic nuclear factor (HNF)3β (FOXA2). (**C**) Representative immunofluorescence staining of hepatic progenitors. Cells express HNF4α, cytokeratin (CK)19, FOXA2 and GATA4. (**D**) Representative immunofluorescence staining of hepatic progenitors. Cells express HNF4α, AFP and ALB. (**E**) Quantitative RT-PCR analysis at day 0,4,11 and 30 of differentiation of hiPSCs. Data are expressed as a percentage of the value obtained for differentiated VAL9 cells. (TIF 6373 kb)

## Abbreviations

A1AT: Alpha-1-anti-trypsin
AFP: Alpha foetoprotein
ALB: Albumin
ALF: Acute liver failure
ALT: Alanine aminotransferase
APAP: Acetaminophen
ASGR: Asialoglycoprotein receptor
AST: Aspartate aminotransferase
BDM: Biliary differentiation media
bFGF: Basic fibroblast growth factor
BSA: Bovine serum albumin
CD81: Cluster of differentiation 81 (tetraspanin 28)
CHIR99021: Glycogen synthase kinase 3 inhibitor
CK: Cytokeratin
CLDN1: Claudin 1
CYP3A4: Cytochrome P450 3A4
DE: Definitive endoderm
EGF: Epidermal growth factor
ELISA: Enzyme-linked immunosorbent assay
EpCAM: Epithelial cell adhesion molecule
FGF: Fibroblast growth factor
GATA4: GATA binding protein 4
GFP: Green fluorescent protein
GMP: Good Manufacturing Practice
HCM: Hepatocyte culture medium
HCV: Hepatitis C virus
hESC: Human embryonic stem cell
HGF: Hepatocyte growth factor
HLC: Hepatocyte-like cell
ICG: Indocyanine green
hiPSC: Human induced pluripotent stem cell
HLC: Hepatocyte-like cell
HNF: Hepatocyte nuclear factor
HPLC/MS: High-performance liquid chromatography tandem mass spectrometry
HPM: HamF12/Williams
IHC: Immunohistochemistry
LY294002: Phosphoinositide-3-kinase inhibitor
MOI: Multiplicity of infection
NANOG: Homeobox transcription factor
OCT4: Octamer-binding transcription factor 4
OPN: Osteopontin
PAS: Periodic acid-Schiff
PBS: Phosphate-buffered saline
PCT: Polymerase chain reaction
SOX: Sex determining region box
TRA-1-60: Tumour resistance antigen 1–60
UGT: UDP glucuronosyltransferase
VAL9-HEP: VAL9 hepatocytes
