# Supplementary material for: Transplantation of hESC-derived hepatocytes protects mice from liver injury
Source: Stem Cell Res Ther. 2015 Dec 12;6:246. doi: 10.1186/s13287-015-0227-6 (PMC4676869; doi:10.1186/s13287-015-0227-6)
Supplement: Additional file 1: Table S1. — Antibodies used in this study. (PDF 48.5 KB) [file 13287_2015_227_MOESM1_ESM.pdf]

Supplementary Table 1 Antibodies used in this study

Antibody	Company	Reference number	Dilution
Primary			
OCT4	Santa Cruz	SC-5279	1:200
NANOG	R&D	AF1997	1:100
TRA-1-60	Millipore	MAB4360	1:150
GATA4	Santa Cruz	SC-1237	1:200
SOX17	R&D	MAB1924	1:150
FOXA2	Santa Cruz	SC-6554	1:100
HNF4α	Santa Cruz	SC-8987	1:200
CK19	Dako	M0888	1:200
HNF6	Santa Cruz	SC-13050	1:200
HNF1α	Santa Cruz	SC-135939	1:200
EPCAM	AbCys	ABC171	1:200
AFP	Santa Cruz	SC-8399	1:200
ALB	Cedarlane	CL2531A	1:200
CYP3A4	Santa Cruz	SC-27639	1:100
A1AT	Dako	A0012	1:400
CLDN1	Life Technologies	187362	1:200
CD81	BD Pharmingen	555675	1:200
SRB1	Novus Biologicals	NB400-131	1:100
OPN	Santa Cruz	SC-21742	1:200
HNF1β	Santa Cruz	SC-7411	1:200
CK7	Dako	M7018	1:200
CK18	Chemicon	CBL185	1:100
CFTR	AbCam	AB2784	1:500
Secondary			
Donkey anti-goat	Molecular Probes	A11055 - A11057	1:1000
Donkey anti-rabbit	Molecular Probes	A32306 - A10042	1:1000
Donkey anti-mouse	Molecular Probes	A21202 - A10037	1:1000
