# Supplementary material for: Transplantation of hESC-derived hepatocytes protects mice from liver injury
Source: Stem Cell Res Ther. 2015 Dec 12;6:246. doi: 10.1186/s13287-015-0227-6 (PMC4676869; doi:10.1186/s13287-015-0227-6)
Supplement: Additional file 2: Table S2. — Primers used for RT-PCR of Human mRNAs. (PDF 79.6 KB) [file 13287_2015_227_MOESM2_ESM.pdf]

Supplementary Table 2

Primers used for RT-PCR of Human mRNAs

Gene	Direction	Primer sequence 5' → 3'	Annealing temperature, °C	Amplicon size, bp
HNF4α	Forward	CGGGTGTCCATACGCATCCTTG	60	347
	Reverse	GACCCTCCCAGCAGCATCTCCT		
AFP	Forward	AGCTTGGTGGTGGATGAAAC	58	248
	Reverse	CCCTCTTCAGCAAAGCAGAC		
ALB	Forward	CCTTTGGCACAATGAAGTGGGTAACC	58	354
	Reverse	CAGCAGTCAGCCATTCACCATAGG		
FOXM1B	Forward	GGGCGCACGGCGGAAGATGAA	63	493
	Reverse	CCACTCTTCCAAGGGAGGGCTC		
LDLR	Forward	GGGCTGGAAATTGCGCTGGACCGTC	60	374
	Reverse	TCACAGACGAACTGCCGAGAGATGC		
β-*ACTIN*	Forward	GCACTCTTCCAGCCTTCCTTCC	60	521
	Reverse	CTGCTGTCACCTTCACCGTTCC		

Primers used for qRT-PCR of Human mRNAs

**Gene**	**Direction**	**Primer sequence 5' → 3'**
OCT-4	Forward	GGTATTCAGCCAAACGACCA
	Reverse	TTGCCTCTCACTCGGTTCTC
HNF-4α	Forward	GCCTACCTCAAAGCCATCAT
	Reverse	GACCCTCCCAGCAGCATC TC
ALBUMIN	Forward	TGAGAAAACGCCAGTAAGTGAC
	Reverse	TGCGAAATCATCCATAACAGC
OTC	Forward	GCTGATTACCTCACGCTCCA
	Reverse	TGGTTACACTAGCATCCGGC
MRP	Forward	GCCGGTGGTCAGATTATCAT
	Reverse	GATCTTGGATTTCCGAAGCA
UGT1A1	Forward	AGACGTACCCTGTGCCATTC
	Reverse	CCGTCAGCATGACATCAAAG
UGT2B7	Forward	TCAGCTTCTCTCCTGGCTACACTT
	Reverse	ACGTCAGCTTTCCCCATTGTCTCAG

CYP1A2	Forward	AACAAGGGACACAACGCTGAAT
	Reverse	GGAAGAGAAACAAGGGCTGAGT
CYP2C9	Forward	CCTCTGGGGCATTATCCATC
	Reverse	ATATTTGCACAGTGAAACATAGGA
CYP2C19	Forward	TTCATGCCTTTCTCAGCAGG
	Reverse	ACAGATAGTGAAATTTGGAC
CYP3A4	Forward	CCTTACATATACACACCCTTTGGAAGT
	Reverse	AGCTCAATGCATGTACAGAATCCCCGGTTA
