# Supplementary figures and images for: Transplantation of hESC-derived hepatocytes protects mice from liver injury
Source: Stem Cell Res Ther. 2015 Dec 12;6:246. doi: 10.1186/s13287-015-0227-6 (PMC4676869; doi:10.1186/s13287-015-0227-6)

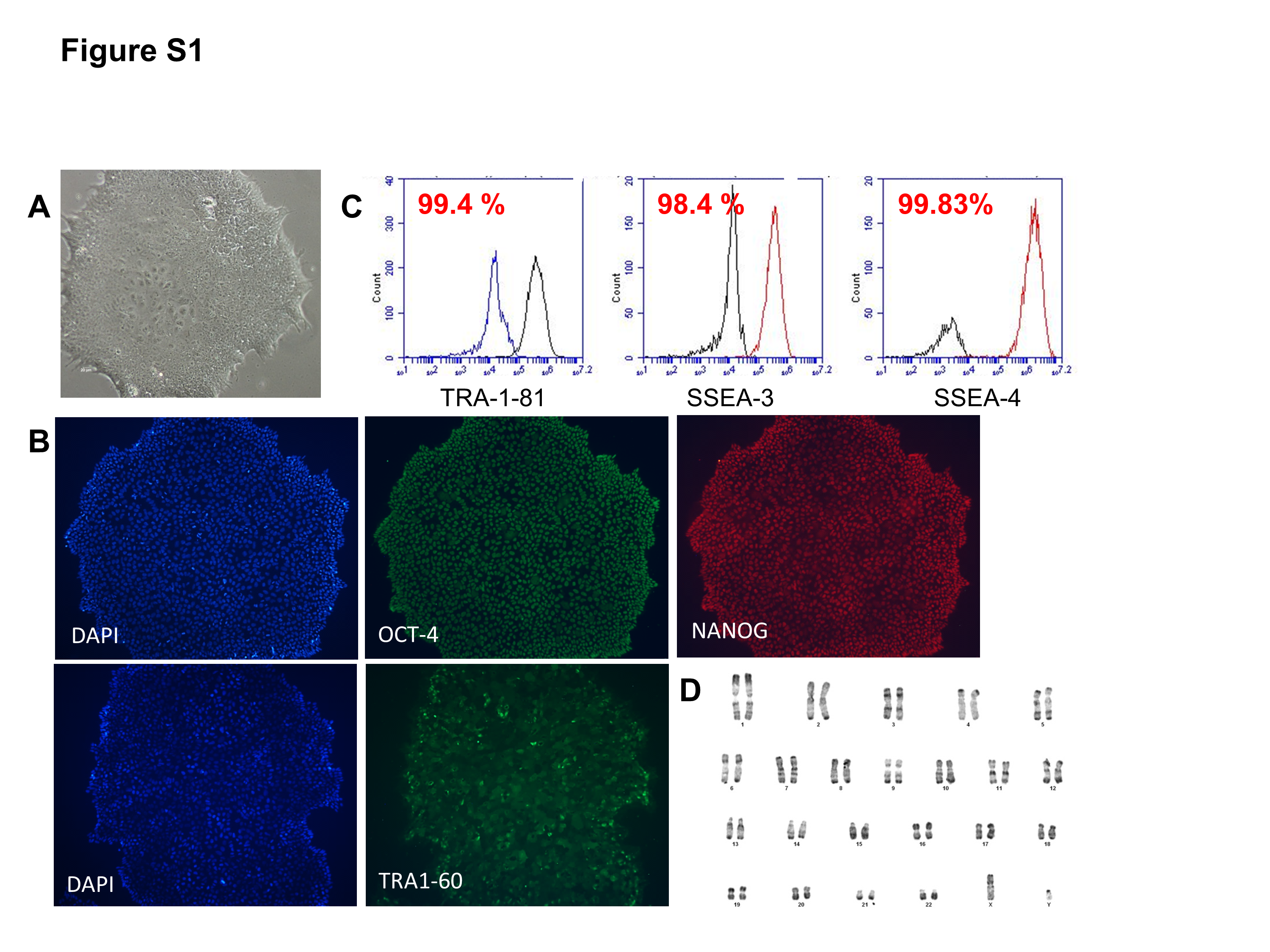

Supplement: Additional file 3: Figure S1. — Pluripotency of VAL9 hESCs. (A) Phase-contrast image of a representative VAL9 hESC colony. (B) Representative immunofluorescence staining for human OCT-4, NANOG and TRA1-60. (C) Fluorescence-activated cell sorting (FACS) analysis of the expression of stem cell specific surface markers: TRA1-81, SSEA-3, SSEA-4. (D) Karyotype analysis of VAL9 hESCs. (TIF 4911 kb) [file 13287_2015_227_MOESM3_ESM.tif]

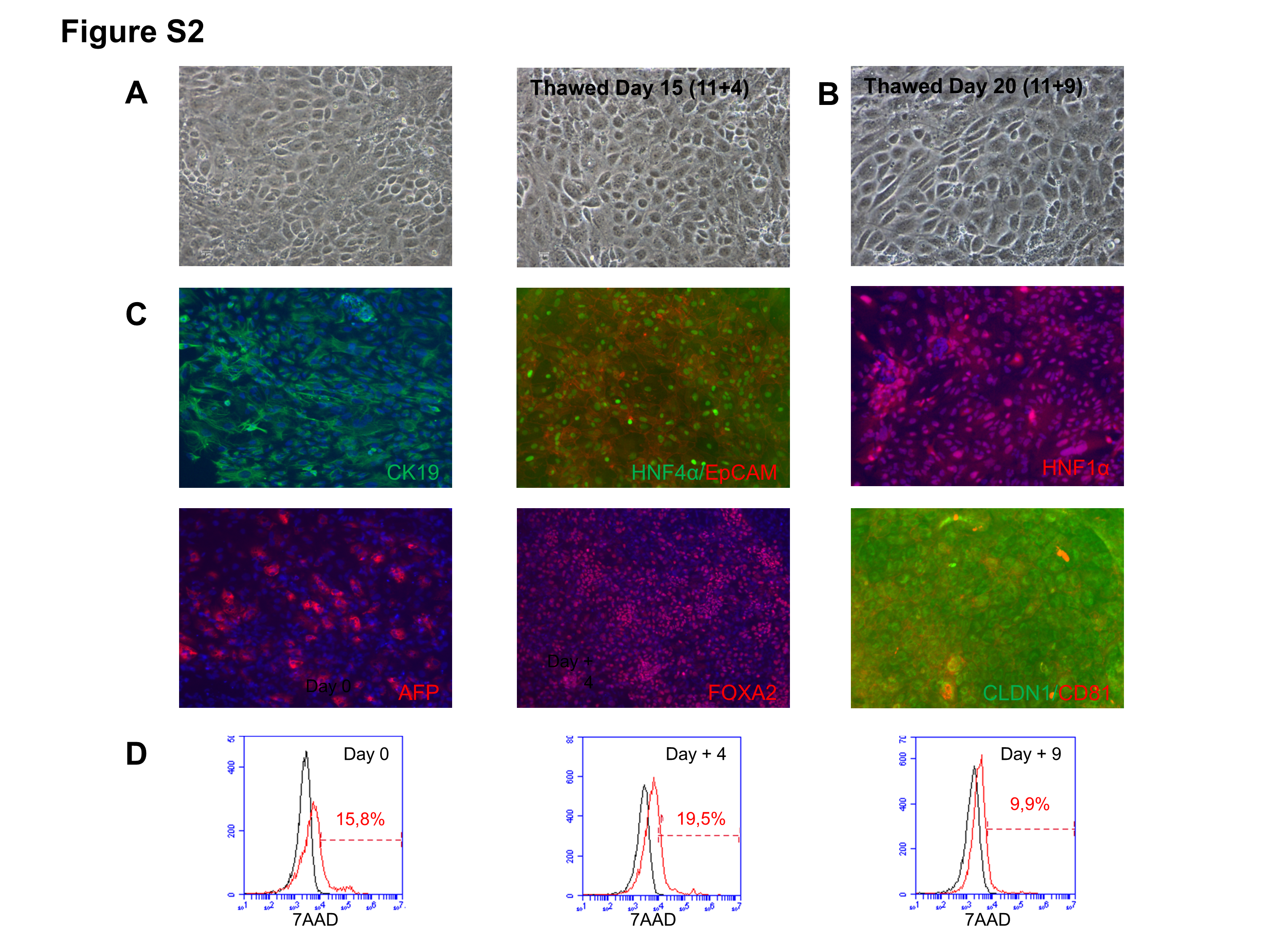

Supplement: Additional file 4: Figure S2. — Cryopreservation of VAL9-hepatoblasts. (A) Phase-contrast image of a representative VAL9-hepatoblasts at day 15 of differentiation before (fresh) and after cryopreservation (4 days after thawing). (B) Phase-contrast image of a representative VAL9-hepatoblasts at day 20 of differentiation (9 days after thawing). (C) Representative immunofluorescence staining for human cytokeratin (CK)19, hepatic nuclear factor (HNF)1α, alpha foetoprotein (AFP), FOXA2 ; and co-staining for human hepatic nuclear factor (HNF)4α/epithelial cell adhesion molecule (EpCAM), and for human claudin (CLDN)1/CD81. (D) Representative fluorescence-activated cell sorting (FACS) analysis for cell viability of thawed VAL9 cells at different time points after thawing as detected by 7AAD staining (TIF 5667 kb) [file 13287_2015_227_MOESM4_ESM.tif]

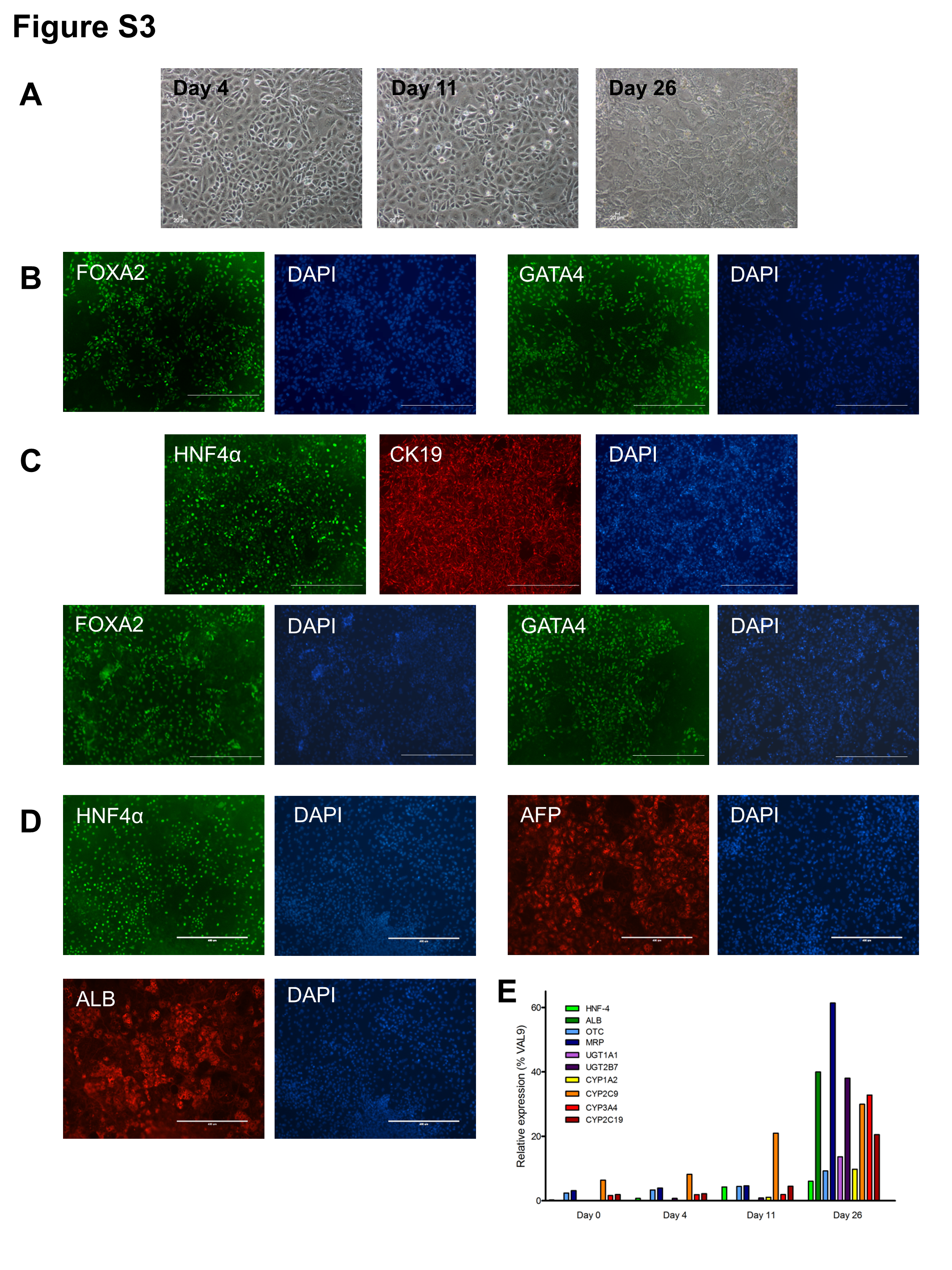

Supplement: Additional file 5: Figure S3. — Differentiation of hiPSCs into hepatocytes. (A) Phase contrast microscopy images of cell morphology at different key steps of the differentiation protocol. (B) Representative immunofluorescence staining of definitive endoderm. Cells express GATA binding protein (GATA)4, and hepatic nuclear factor (HNF)3β (FOXA2). (C) Representative immunofluorescence staining of hepatic progenitors. Cells express HNF4α, cytokeratin (CK)19, FOXA2 and GATA4. (D) Representative immunofluorescence staining of hepatic progenitors. Cells express HNF4α, AFP and ALB. (E) Quantitative RT-PCR analysis at day 0,4,11 and 30 of differentiation of hiPSCs. Data are expressed as a percentage of the value obtained for differentiated VAL9 cells. (TIF 6373 kb) [file 13287_2015_227_MOESM5_ESM.tif]
